# Evaluation of Heavy Metal Content in Feed, Litter, Meat, Meat Products, Liver, and Table Eggs of Chickens

**DOI:** 10.3390/ani10040727

**Published:** 2020-04-22

**Authors:** Mohamed A. Korish, Youssef A. Attia

**Affiliations:** 1 The Strategic Center to Kingdom Vision Realization, King Abdulaziz University, P.O. Box 80200, Jeddah 21589, Saudi Arabia; 2 Arid Land Agriculture Department, Faculty of Meteorology, Environment and Arid Land Agriculture, King Abdulaziz University, P.O. Box 80208, Jeddah 21589, Saudi Arabia

**Keywords:** heavy metals, chicken eggs, meat, meat products

## Abstract

**Simple Summary:**

Foods contain a wide range of trace elements, some of these are of important nutritional value, such as iron (Fe), copper (Cu), zinc (Zn), manganese (Mn), selenium (Se), cobalt (Co), and chromium (Cr), while others have toxic effects, such as lead (Pb), cadmium (Cd), and nickel (Ni). The food chain creates potential health hazards with regards to the transmission of toxic elements to animal tissues, and subsequently to humans. Therefore, the feeding strategy of animals is a useful tool for limiting health hazards through the food chain. We assessed the concentrations of Fe, Cu, Zn, Mn, Se, Co, Cr, Pb, Cd, and Ni in eggs and chicken meat products, as well as those in the feed and litter of chickens, to determine the possible risk posed to consumers from the consumption of chicken products. The results indicated that Cd, Pb, and Se were under detectable levels in chicken meat products and eggs, suggesting that there is no threat from toxic heavy metals. On the other hand, the greatest concentration of heavy metals was recorded in broiler liver except for Cr, Co, and Ni. Meat products exhibited higher Cd, Cu, Mn, Ni, Pb, and Co levels than raw meat and table eggs. Overall, findings indicated that levels of Pb and Ni were four times and seven times high than the tolerable upper limit showing a health threat to humans from the consumption of chicken meat products.

**Abstract:**

We assessed the concentrations of Fe, Cu, Zn, Mn, Se, Co, Cr, Pb, Cd, and Ni in chicken meat and meat products, feed, and litter, as well as laying hens’ eggs, feed and litter to monitor the quality of products on the market and their safety for human consumption as judged by recommended daily allowance (RDA) and tolerable upper levels. Samples were chosen as the most popular poultry products in Saudi Arabia. A total of 45 broiler samples of frozen or fresh meat, liver, burger, or frankfurter were chosen from the same brand. Additionally, 60 table eggs from four commercial brands were collected, and the edible parts of these were used to determine levels of minerals and toxic elements. Furthermore, 30 feed and litter samples were collected from the starter, grower, and layer diets of broilers and laying hens. The results indicated that there were significant levels of most of the trace elements and heavy metals in the different meat sources. Furthermore, the liver contained the highest levels of elements, except for Cr, Co, and Ni. The highest Cr level was detected in the fresh meat, followed by frozen meat. Trace elements (Mn and Co) and heavy metals (Ni and Pb) were not detected in either the frozen or the fresh meat. The chicken burger and the frankfurter exhibited similar trace-element and heavy-metal contents, except for Zn and Mn, as the frankfurter showed higher concentrations than the burger. Differences in most of the trace and toxic elements among the different sources of eggs were not found to be significant, except for Zn. Differences between the broiler meat and table eggs were only substantial for Fe and Zn. Fe was significantly higher in meat than in eggs, and the opposite trend was found for Zn. The liver contained higher heavy metals than the eggs, except for Cr. In addition, the burger had higher concentrations of essential (Cu and Co) and heavy metals (Pb and Ni) than the eggs but had lower levels of Zn and Cr. The frankfurter exhibited significantly higher levels of Fe, Cu, Mn, Co, Pb, and Ni than the eggs but lower levels of Zn and Cr. To summarize, Cd, Pb, As, and Se were not detected in the broiler meat or eggs, indicating no risks from these toxic elements. Conversely, the liver exhibited the highest content of heavy metals, except for Cr, indicating that the intake of Pb and Cd was above the recommended daily allowance (RDA) for adults. The meat products exhibited higher Pb, Cd, and Ni levels than the broiler meat and the table eggs, suggesting that they posed a health threat to humans, and the intake of Pb in the meat products was higher than the RDA. Thus, chicken meat and table eggs, which are primary protein sources, are safe sources of human nutrition, while liver and meat products may present potential health hazards through the food chain.

## 1. Introduction

Poultry products, such as eggs, meat, liver, and meat products (i.e., burgers, luncheon meat, and frankfurters) are worldwide primary sources of protein, energy, vitamins, and minerals because they are nutritious, delicious, and affordable and provide much of the recommended daily allowance (RDA) of trace minerals, proteins, and energy [1,2,3,4,5]. However, poultry products may pose risks due to contamination with heavy metals from the environment or through the food chain [6,7,8]. The diet of broilers is commonly supplemented with microelements such as Fe, Cu, Zn, Mn, I, and As to sustain the growth and health of birds [3,4]. Minerals are also essential for maintaining correct metabolic activity, the balance of bodily functions, and immunity in living organisms [8,9]. However, trace minerals can exceed animal requirements and are consequently excreted in manure, which has a negative environmental impact [9].

In addition, other heavy metals, such as Pb, Cd, and Hg, are not necessary to the integrity and function of the body [10,11]. Generally, increasing the intake of toxic elements poses hazards to both animals and humans [12]. The mineral content of animal products is important for healthy human embryonic development [3,4,13,14] and has been suggested for use as a bioindicator of pollution by environmental elements [15]. However, animal products might have high concentrations of toxic elements that originate mainly from the feed, water, litter, and the environment [9,10]. Cd, As, and Pb are hazardous and can be transmitted through the food chain, having a toxic impact on the health of both animals and humans [16]. Lead can cause metabolic harm as a neurotoxin [16] and can adversely affect renal function and hemopoiesis, as well as the nervous and gastrointestinal systems [17,18]. Diet is a source of contamination by Cd, which originates from various food sources and the environment [17] and is transmitted through the food chain to animals and, consequently, humans, inducing kidney dysfunction, hypertension, and pulmonary and hepatocellular damage [18].

In the available literature, mineral pollution in animal feeds is unavoidable, as they may be contaminated by heavy metals due to environmental pollution and presence in supplements and concentrates or the machinery and equipment used during manufacture. Both in human and animals, liver and kidney are very important for detoxification and execration of toxic elements, and they are thus also the most damaged organs due to an excess of toxic elements in feed or food [15,16,17,18].

The As is stored in animal tissues depending on the type of feed intake [19]; As can induce nausea, headache, and severe gut irritation [20]. Similarly, Cu adversely affects kidney, brain, and liver function and can result in haemolytic crisis when consumed at high levels [21]. The assessment of essential trace minerals and heavy metal levels in poultry products are useful tools with regards to nutritional safety and environmental sustainability. Thus, the aim of the present study is to assess the concentrations of Fe, Cu, Zn, Mn, Se, Co, Cr, Pb, Cd, Ni, B, and Al in poultry diets, litter, eggs, liver, meat, and meat products, which is essential to determine the possible risk posed to consumers by the consumption of poultry products, as well as the environmental impact of the heavy metal content of litter.

## 2. Materials and Methods

The experiment was designed to mentor the trace and toxic elements in poultry products in relation to human wellbeing as judged by RDA and upper tolerable levels. The experimental protocol of this work was approved by the Deanship of Scientific Research (DSR), King Abdulaziz University, Jeddah, Saudi Arabia, under project no. ‘G: 475-375-1440’. The work was carried out according to Royal Decree number M59 in 14/9/1431H and did not involve any living animals and/or human subjects. 

### 2.1. Sample Collection and Analyses

The samples were collected from the retail market in Jeddah City, Saudi Arabia during June–July 2017.

The samples included meat and meat products; thus, a whole broiler carcass, equivalent to 45 samples of fresh (cold) carcasses, was collected to represent three brands locally produced in Saudi Arabia.

In addition, 45 samples of meat products such as burgers, frankfurters, and liver were obtained from the carcasses of the same brands produced locally in Saudi Arabia

The carcasses were from grade A with 1 kg weight class having similar production and expiry dates.

Further, 45 frozen carcasses were obtained from three imported, well-known international brands. The frozen carcasses were imported from Brazil, France and USA.

The carcasses were deboned and skinned, and the meat was cut into two pieces and minced using a meat mincer (Moulinex-HV8, Paris, France).

Additionally, 60 table eggs from four commercial brands (A, B, C, D) were collected. The egg samples were from four different commercial brands produced in Saudi Arabia. The eggs were cracked open, and the edible parts (albumen and yolk) were mixed and homogenized using a Moulinex-HV8 (France).

Fifteen samples of broilers were collected from broiler farms to represent the broiler starter, grower, and finisher commercial standard corn-soybean diets as five samples of each diet. The diets are commercial and consist mainly of corn and soybean meal. Broiler diets meet the nutrient requirements of broiler during different growth periods [22]. The composition of the starter broiler diet was metabolizable energy (ME) 3010 kcal/kg diet, crude protein (CP) 22.3%, methionine + cysteine 0.93%, lysine 1.25%, Ca 1%, and available phosphorus 0.51%. The grower broiler diet contained ME 3150 kcal/kg diet, CP 20.3%, methionine + cysteine 0.88%, lysine 1.20%, Ca 0.90%, and available phosphorus 0.45%. The finisher broiler diet consisted of ME 3260 kcal/kg diet, CP 18.1%, methionine + cysteine 0.73%, lysine 1.10%, Ca 0.83%, and available phosphorus 0.41%.

The same number of samples were collected from layer farms to represent starter, grower, and layer commercial standard diets and consisted mainly of corn and soybean meal as five samples of each diet. The laying diets were a soybean meal-based diet that meets nutrient requirements of white eggshell layers during the starter, grower, and laying periods [22]. The starter layer diet consisted of ME 2918 kcal/kg diet, CP 21.0%, methionine + cysteine 0.81%, lysine 1.11%, Ca 0.96%, and available phosphorus 0.45%. The grower layer diet consisted of ME 2710 kcal/kg diet, CP 15.8%, methionine + cysteine 0.68%, lysine 0.77%, Ca 0.93%, and available phosphorus 0.43%. The laying hens diet contained ME 2800 kcal/kg diet, CP 17.5%, methionine + cysteine 0.74%, lysine 0.85%, Ca 3.7%, and available phosphorus 0.36%.

In addition, 30 samples of litter from broilers and laying hens were collected from the same houses where feed samples were collected in the Jeddah area, Saudi Arabia. Thus, there were five samples of each type of litter of broilers and laying hens.

The rearing, farming, and feeding practices of the broilers and laying hens were in accordance with the standard breeder’s management guide, but these details are not available from the producers. Broilers and laying hens are usually fed according to the breeder guide that meets their nutrient requirements. They were offered ad libitum a pelleted diet and freshwater. The broilers were slaughtered in automatic slaughterhouses according to the Islamic method.

The meat, eggs, diet, and litter samples were dried in a force ventilated oven at 105 °C until a constant weight was achieved. Then, 2 g of each of the dried meat, egg, diet, and litter samples were placed in a flask and digested with a mixture of concentrated HNO_3_ and H_2_SO_4_ (3:1 V/V) [23]. The digestion process was continued until the solution became clear. The samples were then reconstituted in an aqueous matrix containing 0.5% hydrochloric acid and 2% nitric acid, added to stabilize elements as an ionic solution. The samples were transferred to another flask and diluted to 25 mL with distilled water [24]. The trace elements and heavy metals contents were determined using a Varian ICP-Optical Emission Spectrometer (Varian 720-ES, ICP-OEM, Leuven, Belgium).

The working protocols of ICP-MS were adapted from Olajire and Ayodele [25]. The operation condition was Radio Frequency (RF) generator power 1150 W, observation height 12 mm, cooling gas flow 0.7 L/min, 105 auxiliary gas flow 0.5 L/min, analysis pump speed 50 rpm, high purity argon, and detection mass (m/z). The ICP-MS offers auto-dilution techniques to counteract the high total dissolved solids samples. The method has been validated for the determination of Al, As, Cd, Cr, Cu, Fe, Hg, Pb, Mn, Ni, Se, Tl, and Zn in various types of food, including composite diets, cereals, rice, fish and offal by CSL (http://randd.defra.gov.uk/Document.aspx?Document=11372_Appendix5TRACEprocedureformultielementanalysis.pdf).

The daily intake of each essential element and heavy metal in the edible parts of the eggs (yolk and albumen), meat, meat products, and liver was estimated using the concentration of each element in each product and an average of 100 g of the poultry products according to the following equation: daily intake = average consumption of any poultry products (100 g/person/day) × concentration of an element in each product.

The values were compared with the recommended and upper tolerable levels of the Food and Agriculture Organisation (FAO)/ World Health organization (WHO).

### 2.2. Statistical Analysis

The collected data were subjected to a one-way ANOVA using SAS software [26]. The statistical model included the effect of the types of meat or eggs according to the following model:

Yij = μ + Di + eij(1)

where Y = the dependent variables; μ = general mean; D = meat or egg source; and e = random error. Before analysis, all the percentages were subjected to logarithmic transformation (log10 × + 1) to normalize the data distribution. The differences among the means were determined using the Student Newman Keuls test [25]. Significance was considered when the *p* value was 0.05 or less, whereas when the *p* value was between 0.05 and 0.10 it was considered as a trend.

## 3. Results

Table 1 shows the concentrations of essential trace minerals and heavy metals in samples from different diets for broilers. Different broiler diet samples had similar traces of mineral and heavy metals content.

In general, B was detectable in the diets of broiler, but Co and Al were not detected in the diets of the broilers. The B concentrations were higher in the grower and finisher broiler diets than in the starter broiler diets.

Table 2 indicates there were no significant differences among the various diet samples for layers with regards to traces and heavy metals content, except for Zn, which was higher in starter than grower and laying diets.

The highest levels of Pb were from the laying hens’ diet, but the level was 41.4% of the toxic level. The highest levels of As, Ni, and B were found in the growing diet of the layers. Co and Al were under detectable levels in the different types of layers’ diets.

Table 3 displays the levels of essential trace elements and heavy metals of the different litters of the broiler at various stages of production. The litter of the broiler diets showed similar levels of trace elements and heavy metals with the exception of Cu (*p* = 0.027), where the highest level of Cu was during the starter phase. 

The litter of the broilers during the grower phase had the highest Pb level, followed by that of the finisher phase and then that of the starter phase. The highest levels of Cd, As, and Ni were found in the litter of the broilers during the finisher phase.

Co was not delectable in the broiler litter during different stages of production, and Al was detected in the litter only during the starter period, with the grower litter having 2.7 times more than the finisher litter.

Table 4 shows the levels of essential trace elements and heavy metals of different litters of laying hens at various stages of production. The litters showed similar levels of trace elements and heavy metals with the exception of Se (*p* = 0.046), with the highest level of Se noted in the litter of the laying hens and the lowest in the grower litter. In addition, the litter exhibited the highest levels of Cu and Zn. 

The highest levels of heavy metals, such as Cd, As, Ni, B, and Al were found in the litter of the layers during the starter phase. The litter of the laying hens during the grower phase had the highest Pb level, followed by the starter diets.

Table 5 shows the essential trace mineral and heavy metal contents of various chicken meat products and liver. There were significant differences among the various meat sources in most of the trace elements and heavy metals except for Se and As, which were undetectable in all samples. Essential trace elements (Mn and Co) and heavy metals (Pb, Cd, and Ni) were undetectable in the frozen or fresh meat.

The heavy metal (Co, Pb, Cd, and Ni) contents in the liver, burger, and frankfurter were similar. Chicken burger and frankfurter showed similar levels of trace minerals and heavy metals except for Zn and Mn, whereas the frankfurter showed higher concentrations than the burger.

The liver had the highest concentrations of Fe, Cu, Zn, Mn, Pb, and Cd content but not Cr, Co, and Ni. The highest levels of Cr, Co, and Ni were found in fresh meat, burger, and frankfurter, respectively.

Table 6 shows the essential trace element and heavy metal contents of different sources of table eggs. No significant difference in the content of most of the trace elements and heavy metals between various sources of eggs was detected except for Zn, whereas sources B and D exhibited higher concentrations than sources A and C. Essential elements such as Mn, Se, and Co, and heavy metals such as Pb, Cd, As, and Ni, were undetectable in the different sources of table eggs. The Fe content in eggs ranged from 11.83 to 17.70 ppm, while the Cu content ranged from 1.17 to 1.64 ppm.

Table 7 shows essential trace mineral and heavy metal contents of eggs, meat, meat products, and liver. Differences between the element content of broiler meat and table eggs were significant only for Fe and Zn, where the Fe level was significantly higher in meat than in eggs, while the Zn level was lower. In general, the liver had higher levels of toxic elements than the eggs, except for Cr. The burger and frankfurter also had higher concentrations of most of the trace elements than the eggs, except for Cr and Zn. On the other hand, Pb and Ni levels were higher in meat products (burger and frankfurter) than eggs.

The maximum permissible levels of essential elements and heavy metals from the literature in different poultry products are presented in Table 8. The results indicated that the permissible level for each element is different based on the nature of the element and its physiological body function. The maximum was for Zn, followed by B, and the lowest was for Cd.

### Estimated Daily Intake

The estimated daily intake of the essential elements and heavy metals compared with the RDA are presented in Table 9. The consumption of liver can meet the RDA for all trace elements, while contributing a higher intake of Pb and Cd. From a safety point of view, eggs and poultry meat meet the RDA for trace elements and do not present a threat of toxicity from any heavy metals, except Cr. Consumption of meat products, such as burger and frankfurter can supply a considerable part of the RDA of trace minerals and are abundant sources compared with meat and eggs, but they can present a health threat from Pb.

## 4. Discussion

In general, the harmful effects of heavy metals include deleterious functional and physiological effects influencing cell metabolism, having also oxidative impact on biological macromolecules that adversely influence nuclear proteins and DNA [29,48,49,50,51,52,53,54,55,56,57,58,59,60]. These metals are essential to the maintenance of different physiological and biochemical functions in living organisms in small amounts; however, they become harmful when they exceed specific standards, and it is acknowledged that heavy metals can cause cell malfunction and, ultimately, toxicity [29,59]. Sources of contamination by heavy metals are different from one element to another [60,61,62,63,64], and mainly depends on the type of soil, environment risks broadens, animal species and product feeds, and geographic area [65,66,67,68,69].

### 4.1. Essential Elements and Heavy Metals in Frozen and Fresh Meat and Meat Products

It should be mentioned that frozen and fresh meat had similar essential elements and heavy metals contents, except for Cr, which were higher (1.2%) in the fresh meat than frozen meat. It was evident that the type of poultry product had a pronounced effect on the Fe content, and the liver is the most abundant Fe source, according to other authors [43,70]. The values of Fe in different types of meat and liver found herein were higher than those obtained (6.77–7.49) by Elsharawy [70], (41.4–54.9 ppm) by Khan et al. [43] and (12.37–14.39 ppm) by Muhammad et al. [71] for liver and meat from different districts of Pakistan. As with the present results, Alturiqi and Albedair [72] observed that the Fe levels in meat products such as beef loin, pastrami, sausage, and luncheon meat were 175.7, 188.5, 242.4, and 203.1 ppm, respectively. The Fe content of chicken meat from different districts in Saudi Arabia ranged from 135.3 to 290.0 ppm.

The Fe content of frozen and fresh meat, liver, burgers, and frankfurters exceed the RDA for humans while burgers can supply a considerable amount (27.3–30.8% and 84.7–95.3%, respectively) of the RDA of Fe for children and adults, with the liver being the most abundant source. Meanwhile, the values obtained herein for Fe content of varying meat products are in line with those reported by Chowdhury et al. [73] for chicken meat (16.7–60.3 ppm) and meat products (11.4–290.1 ppm). In this context, the liver is the site of metabolism and storage of Fe, which is vital for animal and human nutrition as an essential part of haemoglobin [43,51,71]. Metabolism of proteins, lipids, and carbohydrates is facilitated by Fe, which plays a vital part in the survival and growth of living organisms, cytochrome oxidase, catalase, oxygen-transporting haemoglobin, and myoglobin, as well as the redox process [51]. Deficiency of Fe causes a high susceptibility to gut infections, myocardial infarctions, and nose bleeds [51,70]. The influence of toxic concentrations of Fe in animals includes coma, depression, cardiac arrest, respiratory failure, and convulsions. The maximum tolerable level of Fe for adult females/males (14–70 years old) and children (0–8 years old) is 45 and 40 ppm per day, respectively [41].

The maximum permissible levels for Cu in meat and meat products were reported to be 10 ppm and 1 ppm [35,37]. In addition, the allowable level for Cu in meat and offal in Egypt should not exceed 15 ppm [44]. Thus, fresh and frozen meat, burger, and frankfurter were found to be safe for human consumption and within the permissible level, but the liver samples showed the highest values of Cu and posed a hazard for humans. The Cu content of chicken meat from different districts in Saudi Arabia ranged from 2.30 to 7.88 ppm [72]. In accordance with the current findings, Cu levels in meat products, beef loin, pastrami, sausage, and luncheon meat have been found to be 14.84, 11.11, 18.51, and 13.78 ppm, respectively [73]. Cu is an essential element for various enzymes and is mostly stored in the liver and muscle and is involved in different body functions [22], but an increased Cu dose provokes stomach, nausea, jaundice, diarrhea, and severe colic, and liver and renal problems, as well as anemia, while excessive deposition of Cu in the gizzard, liver, eyes, and brain are characteristic of Wilson’s disease [53,70]. Therefore, the consumption of animal products with increased Cu levels may pose a threat to public health [51,59,70,72].

It was shown that Zn residuals in frozen and fresh meat and meat products are higher than the permissible levels for human consumption (20 ppm) [34], while liver samples showed the highest vales and burgers showed the lowest values. Similarly to the present results, it was found [59,73] that the concentration of Zn in the liver was 22.2–74.84 ppm and was 4.46–168.7 ppm in meat. Furthermore, it was observed [73] that Zn in meat products ranged from 17.2 ppm for burgers to 138.4 for chicken wings. However, [71] found that Zn levels in meat and liver are similar (12.23 vs 13.93, ppm). In addition, it was revealed [43] that Zn levels in the liver, thigh, and breast meat from different districts in Pakistan were similar and ranged from 106.6 to 110.3 ppm. Zinc content in poultry products may vary due to geographical area and the type of product [74,75] for example, the values of residual Zn varied in meat products (30.3–73.9) and in meat (27.9–36.9) among different studies from Saudi districts [72], in Zambia [76], and in Pakistan [77], being similar to the present findings.

The amounts of Mn in frozen and fresh meat and different poultry products recorded herein agree with those noted by others [72,77] for chicken meat (0.0–9.98 ppm), and liver (0.24–4.32 ppm) [34,35]. The liver is the seat of metabolism, and thus, a high concentration of Mn in the liver would be expected. Likewise, previous studies [8,43,78] found that the Mn content in the liver (1.12–340 ppm) was higher than that in the muscle (0.696–102 ppm). In addition, Mn contents in meat products, beef loin, pastrami, sausage, and luncheon meat were 15.73, 11.97, 18.33, and 32.62 ppm, respectively [72]. The Mn value of chicken meat from different districts in Saudi Arabia ranged from 21.48 to 34.42 ppm. The toxic impact of Mn includes a decrease in fetal weight and retardation of the skeleton and internal organs [79], as well as a decrease in birth weight of term-born infants [80]. The toxicity of Mn may cause DNA damage, chromosomal aberrations, and result in a harmful influence on the embryo and fetus [81], due to accumulation in various brain regions [82], neurotoxicity [83], and Parkinson-like syndrome [84,85] and the generation of reactive oxygen species causing oxidative stress [86,87].

In this study, Se was not recorded in frozen and fresh meat, different meat products and liver, showing no residual impact on human health. It is worth mentioning that Se is an essential mineral for selenoproteins, living organisms, and is involved in metabolism, reproduction, immunological responses, and antioxidant balance [55,88,89]. Se was found to be 0.087–0.115 ppm in liver samples, 0.133–0.164 ppm in breast meat, and 0.169–0.200 ppm in thigh meat in [59]. It has been cited that Se levels are significantly higher in the breast muscle and liver of broiler and much higher in the liver than the other tissues [90,91]. However, excess Se may cause harmful effects, for example, nail changes and alopecia [92]. As selenosis progresses, decreased cognitive function, weakness, paralysis, and death can occur [93]. Post mortem, a blood selenium level > 1400 ppm is consistent with acute toxicity as the cause of death during the first day of exposure.

The concentration of Co in frozen and fresh meat was undetectable and was lower than that in the liver, burgers, and frankfurters that had similar amounts (2.22–2.39 ppm), which is markedly higher than in meat. The residual Co in the liver samples indicated that the liver is the site of Co metabolism, whereas the content of Co in meat products, burgers, and frankfurters showed contamination with Co during the manufacturing process. Cobalt is an essential constituent of vitamin B_12_; however, data relating to Co toxicity, to the best of our knowledge, is rare in the literature [105] and was established by [22] to be 100–200 ppm for poultry. The negative health effects of Co include endocrine deficits, neurological syndromes (e.g., visual impairment and hearing), and cardiovascular problems. The adverse health impact of Co does not occur at a Co blood level below 300μg/L in healthy subjects, which is not connected with changes in concentrations of hemoglobin, red blood cell count, and hematocrit, nor with changes in neurological, cardiac, or thyroid function [105,106]

The toxicity of heavy metals negatively affects animal performance and human health through food chain and depends on the type of metals, metal intakes, age and health status of human [94,95]. In this study, differences in Cr contents of fresh and frozen meat were obvious, being higher in fresh than frozen meat. In addition, different meat products and liver had lower Cr than frozen meat. Cr contents of different meat and meat products and liver found herein were higher than tolerable levels, which are 1.0 ppm in meat and 0.5 ppm in liver [34,37]. The application of agriculture technology has resulted in the release of Cr into the environment, causing Cr hazards as a result of sewage, fertilizers, Cr dust, and using wastewater in irrigation, which influences the food chain. As with the present results, Cr levels have been found to be 0–0.69 ppm in chicken meat, while they were 0–4.33 ppm in meat products [73]. In addition, the Cr level was 0.06 ppm in meat and ranged between 0.08 and 0.11 in the liver in three districts in Pakistan [43]. Cr concentration was 0.061–0.111 ppm in meat samples and 0.086–0.092 ppm in liver samples [59]. Furthermore, the Cr concentration range was 0.15 ppm [71] and 0.064–0.073 ppm in the liver and 0.075 ppm in muscle [95]. It is worth mentioning that improvements in insulin sensitivity, blood glucose, insulin, lipids, hemoglobin, lean body mass, and related variables are seen in response to improved Cr nutrition [96,97], and further evidence was exhibited in broilers where Cr supplementation decreased the blood glucose level [98]. Cr toxicity increases reactive oxygen species [50,99,100,101,102,103] damaging proteins and DNA [104]. This is dependent on the type of Cr; Cr (VI) is considered to be carcinogenic and can cause problems in the liver, kidneys, neural tissues, and the circulatory system. Skin irritations and ulcers can also occur, as well as metabolic defects such as diabetes and heart problems [51,100,101,102].

Frozen and fresh meat was free of Pb residual in the present study, but Pb in meat products and liver was similar and exceeded the tolerable upper intake [43]. The permissible limit for lead residues in meat and offal must not exceed 0.1 ppm for meat and liver [34,44]. Several researchers have reported similar values of Pb residuals in chicken meat, (0–3.94, ppm) [73]. In addition, the Pb concentrations in the flesh and liver samples were 0.25–0.26 and 0.31 ppm, respectively, being higher in the liver than the meat [70]. In this respect, Pb concentration ranged from 0.09–0.51 ppm in the broiler meat [94]. In addition, Pb residuals in chicken meat in different Saudi Arabia districts were in the range of 7.61–10.49 ppm [72], 0.055–0.116 ppm in meat and 0.068–0.093 ppm in the liver [59], 0.07 in meat and 0.056–4.15 ppm in the chickens’ liver [71,76], and from 0–2.23 ppm in meat and 0–7.56 ppm in the liver [43,77].

Lead bio-accumulates in animal and human tissues, mainly in the liver and the bones leading to several diseases such as irritability, cardiovascular problems, auditory, neuropathy, wrist, and food drop, haemolytic anemia, atherosclerosis, and liver apoptosis [50,51]. The nervous, hematopoietic, and adrenal systems are the main systems that are sensitive to Pb toxicity [9,43]. Lead is one of the riskiest heavy metals when consumed through the food chain, and Pb has marked side effects on human health since it is transmitted through the food chain; nonetheless, it is not indispensable for biological function [50,59]. Heavy metals contaminations such as lead contamination can result from the use of foods like vegetables, meat, fruits, seafood, wine herbicides, chemical fertilizers, as well as the use of sewage resulting in soil and environmental pollution and is represented as biological biomarkers [53,72,94,107,108,109].

The results of Cd levels in frozen and fresh meat, meat products, and liver recorded herein agree with those reported in chicken meat (1.36–1.68 ppm) and meat products (3.06–4.08 ppm) [72]. The results showed that frozen and fresh meat is safer than meat products (0.379–0.438 ppm) and liver (1.12 ppm). The permissible limit for Cd in poultry meat and offal was determined to be 5 ppm for meat and 20 ppm for poultry offal [44]. According to these limits, most tested samples of meat products, except for the liver, were within the allowable levels and considered safe for human consumption. Additionally, the permissible limit for Cd in meat and liver was reported to be 0.05 ppm [34,37]. The value of Cd in the liver found herein represents a hazard level according to [107], which is estimated to be 1 ppm as the tolerable upper level, and the estimation of 0.06–0.07 ppm [43].

Similarly to the present findings, Cd has been found to be higher in the liver than in thigh meat, and the latter was higher than in breast meat [70,108]. The values of Cd published in the literature ranged from 0.001–0.002 ppm in meat [59], 0.006–0.23 ppm in different meat products [73], and 0.002–1.6 ppm in the liver [59,71,77,109]. Cadmium is chiefly found in the earth’s crust and is easily absorbed by the organic substances that form the soil, thereby presenting a high risk due to transportation through the food chain from the earth, to food and animals and/or humans [94]. In addition, contamination of Cd can result from chemical fertilizers, particularly phosphate, in the soil, lakes, and groundwater supplies and can negatively impact animals and fish through the food chain [59,73]. In addition, Cd has greater adverse effects on children in whom Cd accumulates to a greater extent in the tissues than the adults. Cadmium is a dispensable metal, but increasing Cd intake above the tolerable level causes respiratory symptoms and lung damage, renal dysfunction, hepatic injury, hypertension [43,109], mental retardation, cardiovascular and auditory systems dysfunction [50,77], carcinogenesis, and mutagenesis diseases [51,76]. The most important negative impact of Cd toxicity is Itai-Itai disease in humans, which directly interferes with calcium and bone mineralization resulting in osteoporosis and osteomalacia [52,110]. Cd causes significant alterations in the detoxification of enzymes in the gizzard and liver [52].

Arsenic is a metalloid, which indicates that it has both non-metallic and metallic characteristics [94]. The residual of As was not observed herein in different meat samples, meat products, and liver, showing the safety of different chicken protein products. In other studies, As was recorded at 0–0.01 ppm in meat and meat products [73]. The values of As in meat and liver were 0.36–0.49 ppm and 0.77 ppm, respectively [70,111]. Other researchers obtained As values of 0.012–0.029 ppm in meat and 0.023–0.049 ppm in the liver [52,59]. Additionally, As levels were 0.003–0.09 ppm in various meats of chicken [94]. The permissible limit for As residues in poultry meat and offal has not yet been set, according to [44,70]; however, the allowable limit for As in poultry meat and offal was estimated to be 0.1 ppm for meat [39]. The contamination of the environment results from the chemical and glass industries, and the pollutants reach water resources, where they come into contact with marine life. As could enter the environment and water resources as a result of As application in medicine and livestock production [50,51]. The accumulation of As in meat is low, and the principal tissues involved in accumulation are the gizzards and the liver [70]. As is the main cause of acute heavy metal poisoning in adults. Exposure to As can induce liver disease, cardiovascular problems, diabetes, cancer, and skin disease.

There was no risk assessed for frozen and fresh meat and meat products due to Ni contamination in this study. The permissible limit for Ni in poultry meat was estimated to be 0.1 ppm in [39]. However, Ni was recorded at 0.057–0.106 ppm in meat and 0.003–0.277 ppm in the liver in [95], 0.036–0.069 ppm in meat and 0.051–0.059 ppm in the liver in [59], and 0.13 ppm in muscle and not noticeable in the liver in [71]. Nickel is an essential element for red blood cell formation, although when excess Ni enters the body via ingestion, inhalation, or absorption, Ni toxicity can be observed and affects foetal organs such as the larynx, nose, and lungs, and can also alter the heart and the prostate [71,95]. It seems that Ni toxicity or contamination only results from very high consumption of Ni, mainly when feeds and/or foods are cultivated in Ni-rich soils, thus contributing greater quantities of Ni to the food chain [59,95].

Frozen and fresh meat and different meat products and liver were free from B residual in the current study. The literature values of B in different poultry products are absent, and this is the first time that B levels in poultry meat, meat products, and liver have been reported. The permissible limit for B in poultry meat and offal was estimated to be 10 ppm for meat and eggs in [112]. The lethal dose of boric acid in one-day-old chicks was found to be 2.95 +/− 0.35 g/kg of body weight, which classifies this product as only slightly toxic to chickens [113]. Boron residue levels in the brain, kidney, liver, and white muscle were not significantly increased following a 15-day exposure period to 500 ppm or 1250 ppm boric acid in feed ad libitum chickens for three weeks; however, B markedly increased due to feeding with 2500 ppm or 5000 ppm boric acid. Boron was not accumulated in the soft tissues of the animals but did accumulate in the bone. Normal levels of B in soft tissues, urine, and blood generally ranged from less than 0.05 ppm to no more than 10 ppm [114]. In poisoning incidents, the amount of boric acid in the brain and the liver tissue has been found to be as high as 2000 ppm. Boron may contribute to decreased male fertility in rodents fed 9000 ppm of boric acid in feed [115]. Within a few days, B levels in the blood and most soft tissues quickly reached a plateau of about 15 ppm. Boron in bone did not appear to plateau, reaching 47 ppm after seven days on a diet. Cessation of exposure to dietary B resulted in a rapid drop in bone B [114]. B does not seem to be metabolized in humans and animals, owing to the massive energy needed for the breakdown of the B–O bond [115].

In the present study, the residual of Al was not recorded in the frozen and fresh meat and different meat products and liver. The permissible limit for Al residues in poultry meat is 1 ppm, according to [40]. Conclusively, there was no risk assessed for meat and meat products due to Al continuation in this study. Al interferes with most physical and cellular processes [116]. Al toxicity presents a threat to humans, animals, and plants and results in many diseases [117]. The toxicity of Al might be produced from the interaction between Al and the plasma membrane, affecting most physical and cellular processes in organisms [118]. In humans, Al^3+^ has been shown to replace Mg^2+^ and Fe^3+^ and resulted in disturbances of cellular growth and intercellular communication, as well as in neurotoxicity effects, and secretory functions [119]. The modifications that are induced in neurons by Al are similar to the degenerative lesions observed in Alzheimer patients [51,120].

### 4.2. Essential Elements and Heavy Metals in Eggs

From a risk assessment point of view, eggs were found to be the safest product for trace elements and heavy metals, and these agree with previous findings [43,69]. The average values of iron in different egg sources was in the range of 11.83–12.70 ppm, indicating that eggs are a rich source of Fe. The values observed herein are higher than those found in egg albumen (1.05–1.27 ppm) and yolk (3.19–3.36 ppm) and in eggs (1.47–2.03 ppm) [46,70].

The values of Cu contents of eggs of different sources in the present study were in the range of 1.17–1.64 ppm and were found to be safe for human consumption and within the permissible level. The maximum permissible level for Cu in eggs is reported to be 10 ppm [34,35]. The Cu content in eggs was in the range of 0.009–0.014 ppm in commercial tables eggs in Saudi Arabia [69].

The source of eggs markedly affects eggs’ Zn content; the values ranged from 58.6 to 68.3 ppm with 14.2% difference. In literature, Zn levels found in egg albumen (1.97–2.05 ppm) and yolk (39.9–40.4 ppm) were similar in different districts of Pakistan; however, yolk had a higher Zn content than the albumen, according to Guyot and Nys [121], which indicates that egg yolk is the major contributor to iron and zinc supply. The Zn residual in eggs was in the range of 1–1.13 ppm in commercial tables eggs in Saudi Arabia [69]. Thus, it might be shown that Zn residuals in eggs found herein are higher than the permissible levels for human consumption [43]. Zn as an essential element is crucial for health at an appropriate level for appetite, taste, and smell, immunity, wound healing, and skin health [51]. Zinc deficiency delays the development of sex organs and causes retarded growth in young men [75]. Sources of Zn pollution include mining, purifying of Zn, Pb, and Cd ores, coal burning, steel production, and waste burning [75].

The Mn residuals in different sources of table eggs were absent, suggesting that eggs are safe for human consumption with regards to residual Mn. The tolerable upper level of Mn for eggs is also absent in the literature. The average Mn in the albumen and yolk of eggs was significantly different and was in the range of 0.19–0.31 ppm for albumen and 1.33–1.40 ppm for yolk [43].

The present results indicated a lack of Se residuals in eggs and thus no risk associated with their consumption. The maximum permissible concentration of Se in eggs is 0.5 ppm [112]. Similar to the present results, Se was not recorded in three sources of commercial eggs in Jeddah City in Saudi Arabia [69]. The importance of Se for human consumption and its deterioration effects were previously discussed in the abovementioned section.

Cr in eggs was in the range of 7.96–8.62 ppm with no differences among the four sources of table eggs, but Cr residuals exceeded the permissible concentrations (1 ppm) [34], being higher than those of the meat products and liver, which presented lower levels of risk than the eggs.

The levels of Co in table eggs are below dangerous levels for human consumption. The residual cobalt in different sources of table eggs was absent, confirming the safety of eggs for human consumption.

The concentration of Pb in different sources of table eggs was below the risk level for human consumption, which is 0.5 ppm [37]. In addition, Khan et al. [43] reported that the upper tolerance level of Pb is 0.43 ppm. In this respect, several researchers have reported similar values of Pb residuals in eggs (0.34–12.1, ppm) [73]. The egg albumen had higher Pb content (0.12–0.13, ppm) than yolk (0.06–0.09, ppm) and was lower than that in meat.

There were no Cd residues in eggs, which were below the risk level for human consumption, which is 0.05 ppm [34,37]. The absence of Cd in eggs agrees with the results reported by [77]. The values of Cd published in the literature ranged from 0 to 0.99 ppm in egg albumen and yolk [73]. Furthermore, the Cd level in eggs ranged from 0.51 to 0.68 ppm, and from 0.03 to 0.06 ppm in egg albumen and egg yolk from three districts in Pakistan [43]. The results showed the eggs are safer than meat products (0.379–0.438 ppm) and liver (1.12 ppm).

The level of arsenic in different egg sources was under detectable levels, suggesting there was no risk assessed for eggs due to As contamination. The permissible limit for As residues in eggs has not yet been set, according to [44,70]; however, the allowable limit for As in poultry eggs was estimated to be 0.1 ppm [39]. In addition, As was found to be at 0–0.01 ppm in eggs [73]; furthermore, As concentration was 0.01, 0.01, and 0.004 ppm in albumen, yolk, and whole eggs, respectively [94].

It was found that different sources of eggs showed no Ni residuals and thus are free of Ni contamination. Thus, there was no risk assessed due to B continuation in this study. The literature values of B in eggs are absent, and this is the first time that B levels in commercial eggs have been reported. The permissible limit for B in eggs was estimated to be 10 ppm [112]. The level of Al in eggs was under detectable levels. The permissible limit for Al residues in poultry eggs is 1 ppm, according to [40]. Conclusively, there was no risk assessed for eggs due to Al continuation in this study.

### 4.3. Heavy Metals in Poultry Diets and Litter

The results indicate that trace minerals in broilers’ diets are in general agreement with recommended levels for broilers and layers during different ages [22]. The heavy metals were also below the toxic levels [22]. Essential minerals are important for the normal physiological functions of animals, and several minerals (Fe, Zn, Se) have been used recently in the production of functional foods for human health benefits [13,14]. It should be mentioned that differences in essential trace minerals found herein among different types of diets, and/or between the diets of broilers and layers, regardless of the feeding stage, are acceptable based on the differences in mineral content of different foodstuffs, diet composition, and/or type of mineral premix used, as well as the source of feeds.

Most of the heavy metals were recorded in diet samples used in this study except for Al, but the levels were found to be less than the toxic limit [22], showing the high quality of feeds and that there are few hazards of heavy metals toxicity. Compared with the results reported by Okoye et al. [62], the concentrations of heavy metals recorded herein were less than those for Pb, As, Cd, Cr, Ni, and Cu and were 0.12–0.293, 0.068–0.167, 0.281–0.379, 0.082–0.212, 0.039–0.172, and 0.069–0.205 ppm, respectively, suggesting low pollution by heavy metals in the present study. The toxic concentrations of heavy metals depend on the chemical form of these elements, the poultry species, and the age of the birds; hence, they are the subject of investigation [22,61].

The concentrations of heavy metals, such as Cr and Pb, in broiler and laying hen diets ranged from 1.51 to 5.20 ppm and 2.09 to 5.27 ppm, respectively. The upper tolerable levels of Cr and Pb were suggested as 10–300 and 10–1000 ppm, respectively. This indicates that Cr and Pb are present at alarming levels in the diets tested in the present research. The Pb contamination in feeds found here is mostly related to environmental pollution resulted from fuel brun herbicides, chemical fertilizers, and sewage causing soil and feed contamination [50,51,53,56]. The higher Cr contents in the feed mixture could be due to the use of fertilizers, sewage, Cr dust, and wastewater in irrigation of crops in Saudi Arabia due to limited water resources. In addition, Jeddah city is an industrial and urban area. This could influence the food chain and, thus, the production of reactive oxygen species [50,63,64]. The high Pb and Cr in the feed mixture concurred with increased contents of Pb and Cr in poultry products, which implies health concerns for humans. This is suggesting regular checking of heavy metals to monitor the food safety should be mandatory in this area of the research. Additionally, a correlation of a durable nature has been recorded between heavy metals in diet and excreted waste, which indicates that the poultry diet has the potential to contaminate several components of the environment [32]. Heavy metals may enter the production system of livestock by different routes, including land application of inorganic fertilizers, atmospheric deposition, agrochemicals, animal waste, and biosolids [65,66]. Furthermore, fish and poultry diets have shown various heavy metal content (e.g., Cd, Cr, and Pb), suggesting that the supply of feedstuffs requires greater governmental quality control [67].

In general, trace minerals and toxic elements in the litter of broilers and layers may reflect to some extent the types of feeds, stages of production, and/or types of chickens. These levels are reflected in the diets with some exceptions, particularly for Al, which was not found in feeds but was recorded in the litter, except for in the broiler starter phase, showing that Al is a litter contaminant at higher concentration in layers than in broilers.

It should be mentioned that except for Se, levels of essential minerals and toxic elements in poultry litters were lower than the values reported in the research of several authors [27,28,29,30,31,32,33,34]. Selenium contents in animals’ feeds are influenced by soil Se content and Se supplementation to meet animal requirements [56]. Furthermore, increasing the Se content of animal diets was recently used for the production of functional food such as eggs, milk, and meat [15]. Heavy metals in animal manure lead to the accumulation of toxic elements in the soil and water due to the use of litter as a fertilizer and for soil amendments [67,68] and hence can be transmitted through feed chain to humans.

## 5. Conclusions

It can be concluded that Cd, Pb, As, and Se were not detected in chicken meat and eggs locally produced or imported, indicating a lack of hazards from these toxic elements for humans. However, the liver showed the highest concentration of heavy metals, except for Cr, and the intake of Pb and Cd from broilers’ liver was above the RDA for adults. Meat products, such as burgers and frankfurters, showed higher Pb, Cd, and Ni concentrations than chicken meat and table eggs suggesting a possible health threat to humans. Thus, to improve the quality of poultry products for human consumption, appropriate legislation is required for monitoring the quality of poultry products, as well as the feeds/food and litter of chickens. Additionally, essential measurements should be used for detoxification of heavy metals from the waste. The relationship between the minerals within poultry production and between poultry diets and poultry litter remains fertile for further research.

## Figures and Tables

**Table 1:** Essential trace element and heavy metal content of broiler diets on a dry matter basis compared with the maximum permitted concentration element.

Elements	Broiler Diets			Toxic Level [22]	RMSE	*p* Value
	Starter	Grower	Finisher			
Essential trace elements, ppm						
Iron	142.9	120.3	132.8	4500	58.2	0.764
Copper	11.93	8.94	11.47	50–806	3.16	0.562
Zinc	33.2	30.2	39.2	500–4000	4.89	0.211
Manganese	31.8	27.3	32.6	4000–4800	7.59	0.649
Selenium	2.30	2.21	0.861	5–20	1.85	0.577
Chromium	2.07	4.33	3.19	10–300	2.63	0.113
Cobalt	UDL	UDL	UDL	100–200	ND	ND
Heavy metals, ppm						
Lead	2.09	2.63	5.27	10–1000	3.09	0.136
Cadmium	0.097	0.110	0.111	12–40	0.077	0.077
Arsenic	2.76	2.98	1.48	100	3.29	0.829
Nickel	1.76	2.15	1.46	300–500	1.39	0.088
Boron	4.88	5.56	5.73	200–5000	3.62	0.394
Aluminium	UDL	UDL	UDL	500–3000	ND	ND

RMSE = root mean square error. MPC = maximum permitted concentration, UDL = undetectable level. ND = not done.

**Table 2:** Essential trace element and heavy metal content of layer chickens’ diets on a dry matter basis compared with the maximum permitted concentration element.

Elements	Layer Diets			Toxic Level [22]	RMSE	*p* Value
	Starter	Grower	Layers			
Essential trace elements, ppm						
Iron	99.4	153.7	183.3	4500	67.5	0.774
Copper	9.05	11.14	16.61	50–806	7.32	0.983
Zinc	60.3 ^a^	49.9 ^b^	45.4 ^b^	500–4000	10.8	0.001
Manganese	28.6	32.1	42.5	4000–4800	7.68	0.554
Selenium	2.11	2.86	4.24	5–20	1.48	0.515
Chromium	2.63	1.51	5.20	10–300	1.47	0.166
Cobalt	UDL	UDL	UDL	100–200	ND	ND
Heavy metals, ppm						
Lead	3.02	3.50	4.14	10–1000	5.05	0.181
Cadmium	0.055	0.061	0.096	12–40	0.136	0.373
Arsenic	2.03	3.13	2.78	100	2.36	0.986
Nickel	4.23	9.85	2.18	300–500	2.74	0.187
Boron	6.05	6.41	5.73	200–5000	3.47	0.802
Aluminium	UDL	UDL	UDL	500–3000	ND	ND

^a, b^ means with different superscripts within a row are significantly different. RMSE = root mean square error. MPC = maximum permitted concentration, UDL = undetectable level. ND = not done.

**Table 3:** Essential trace elements and heavy metals contents of the litter of broiler chickens on a dry matter basis compared with values cited in the literature.

Element	Broiler Litter			Literature Values	RMSE	*p* Value
	Starter	Grower	Finisher			
Essential trace elements, ppm						
Iron	581.9	536.4	572.3	852 [27]	66.9	0.180
Copper	28.5 ^a^	23.3 ^b^	24.5 ^a,b^	31.8–335 [28,29,30]	4.25	0.027
Zinc	134.8	99.4	112.3	196–845.1 [28,29]	43.5	0.641
Manganese	241.9	297.7	263.2	375 [27]	235.4	0.996
Selenium	0.00	0.230	0.315	1.1 [31]	0.317	0.955
Chromium	17.1	1.74	3.54	1.5–143 [28,30,32]	7.61	0.159
Cobalt	UDL	UDL	UDL	0.39 [33]	ND	ND
Toxic metals, ppm						
Lead	2.56	4.42	3.75	0.42–107.1 [28,29,30]	5.77	0.729
Cadmium	0.137	0.155	0.172	0.031–19 [28,30,33]	0.251	0.736
Arsenic	UDL	0.0003	0.0005	1.8–38 [30,31]	0.0003	0.123
Nickel	46.7	23.0	54.3	1.71–276 [29,32,33]	29.9	0.502
Boron	UDL	0.238	0.172	NA	0.274	0.851
Aluminium	UDL	3.134	1.153	NA	0.0049	0.812

^a, b^ means with different superscripts within a row are significantly different. RMSE = root mean square error, UDL = undetectable level, ND = not done, NA = not available.

**Table 4:** Essential trace elements and heavy metals contents of the litter of layer chickens on a dry matter basis compared with values cited in the literature.

Element	Layer Litter			Literature Values	RMSE	*p* Value
	Starter	Grower	Laying Hens			
Essential trace elements, ppm						
Iron	617.4	719.1	676.5	852 [27]	60.6	0.291
Copper	27.8	30.8	36.6	31.8–335 [28,29,30]	10.5	0.961
Zinc	90.6	84.0	275.3	196–845.1 [28,29]	77.3	0.679
Manganese	122.3	141.0	141.2	375 [27]	8.41	0.129
Selenium	0.615 ^a,b^	0.451 ^b^	0.691 ^a^	1.1 [31]	0.087	0.046
Chromium	15.2	13.6	10.9	1.5–143 [28,30,32]	2.11	0.288
Cobalt	UDL	UDL	UDL	0.39 [33]	ND	ND
Toxic metals, ppm						
Lead	9.21	11.77	4.53	0.42–107.1 [28,29,30]	4.49	0.409
Cadmium	0.629	0.388	0.278	0.031–19 [28,30,33]	0.27	0.605
Arsenic	0.00071	0.00048	0.00067	1.8–38 [30,31]	0.0003	0.978
Nickel	11.4	10.8	8.74	1.71–276 [29,32,33]	4.71	0.873
Boron	0.461	0.348	0.377	NA	0.057	0.175
Aluminium	7.29	4.27	4.06	NA	0.004	0.608

^ab^ means with different superscripts within a row are significantly different. RMSE = root mean square error, UDL = undetectable level, ND = not done, NA = not available.

**Table 5:** Heavy metal contents of broiler meat, liver, burger, and frankfurter on a dry matter basis in the retail market.

Element	Broilers’ Meat		Chicken Meat Products and Liver			RMSE	*p* Value
	Frozen	Fresh	Burger	Frankfurter	Liver		
Essential trace elements, ppm							
Iron	87.8 ^b^	63.1 ^b,c^	38.1 ^c^	72.8 ^b,c^	288.2 ^a^	45.2	0.001
Copper	0.036 ^c^	0.056 ^c^	6.66 ^b^	7.80 ^b^	19.24 ^a^	2.61	0.001
Zinc	35.8 ^b^	41.4 ^b^	14.9 ^d^	26.4 ^c^	79.8 ^a^	10.57	0.001
Manganese	UDL ^d^	UDL ^d^	6.41 ^c^	9.77 ^b^	18.2 ^a^	3.40	0.001
Selenium	UDL	UDL	UDL	UDL	UDL	ND	ND
Chromium	7.01 ^b^	9.75 ^a^	2.54 ^c^	2.46 ^c^	2.79 ^c^	2.38	0.001
Cobalt	UDL ^b^	UDL ^b^	2.72 ^a^	2.22 ^a^	2.39 ^a^	0.761	0.001
Heavy metals, ppm							
Lead	UDL ^b^	UDL ^b^	16.69 ^a^	14.84 ^a^	16.51 ^a^	4.84	0.001
Cadmium	UDL ^b^	UDL ^b^	0.433 ^a,b^	0.379 ^a,b^	1.12 ^a^	0.763	0.003
Arsenic	UDL	UDL	UDL	UDL	UDL	ND	ND
Nickel	UDL ^b^	UDL ^b^	7.10 ^a^	7.28 ^a^	6.68 ^a^	3.04	0.001
Boron	UDL	UDL	UDL	UDL	UDL	ND	ND
Aluminium	UDL	UDL	UDL	UDL	UDL	ND	ND

^a, b, c, d^ means with different superscripts within a row are significantly different, RMSE = root means square error, UDL = undetectable level, ND = not done.

**Table 6:** Essential trace elements and heavy metals contents of whole eggs on a dry matter basis of different commercial brands in the retail market.

Element	Egg Source				RMSE	*p* Value
	A	B	C	D		
Essential trace elements, ppm						
Iron	11.83	12.70	12.22	12.51	1.10	0.638
Copper	1.17	1.64	1.56	1.61	1.50	0.748
Zinc	58.6 ^b^	64.7 ^a^	60.2 ^b^	68.3 ^a^	3.18	0.001
Manganese	UDL	UDL	UDL	UDL	ND	ND
Selenium	UDL	UDL	UDL	UDL	ND	ND
Chromium	8.04	8.62	7.96	8.25	0.564	0.293
Cobalt	UDL	UDL	UDL	UDL	ND	ND
Heavy metals, ppm						
Lead	UDL	UDL	UDL	UDL	ND	ND
Cadmium	UDL	UDL	UDL	UDL	ND	ND
Arsenic	UDL	UDL	UDL	UDL	ND	ND
Nickel	UDL	UDL	UDL	UDL	ND	ND
Boron	UDL	UDL	UDL	UDL	ND	ND
Aluminium	UDL	UDL	UDL	UDL	ND	ND

^a, b^ means with different superscripts with a row in similar treatment groups are significantly different, RMSE = root means square error, UDL = undetectable level, ND = not done.

**Table 7:** Heavy metals contents of commercial table eggs and broiler meat, burger, frankfurter, and liver on a dry matter basis in the retail market.

Element	Types of Poultry Products					RMSE	*p* Value
	Eggs	Meat	Burger	Frankfurter	Liver		
Essential trace elements, ppm							
Iron	12.3 ^c^	75.9 ^b^	38.1 ^c^	72.8 ^b^	288.2 ^a^	39.4	0.001
Copper	1.49 ^c^	UDL ^c^	6.66 ^b^	7.80 ^b^	19.24 ^a^	2.27	0.001
Zinc	62.9 ^b^	38.5 ^c^	14.9 ^e^	26.4 ^d^	79.8 ^a^	9.51	0.001
Manganese	UDL ^c^	UDL ^c^	6.41 ^c^	9.77 ^b^	18.2 ^a^	2.91	0.001
Selenium	UDL	UDL	UDL	UDL	UDL	ND	ND
Chromium	8.22 ^a^	8.32 ^a^	2.54 ^b^	2.46 ^b^	2.79 ^b^	2.22	0.001
Cobalt	UDL ^b^	UDL ^b^	2.72 ^a^	2.22 ^a^	2.39 ^a^	0.651	0.001
Heavy metals, ppm							
Lead	UDL ^b^	UDL ^b^	16.69 ^a^	14.84 ^a^	16.52 ^a^	4.16	0.001
Cadmium	UDL ^b^	UDL ^b^	0.433 ^b^	0.379 ^b^	1.12 ^a^	0.655	0.001
Arsenic	UDL	UDL	UDL	UDL	UDL	ND	ND
Nickel	UDL ^b^	UDL ^b^	7.10 ^a^	7.28 ^a^	6.68 ^a^	2.61	0.001
Boron	UDL	UDL	UDL	UDL	UDL	ND	ND
Aluminium	UDL	UDL	UDL	UDL	UDL	ND	ND

^a, b, c, d,e^ means with different superscripts within a row are significantly different, RMSE = root means square error, UDL = undetectable level, ND= not done.

**Table 8:** The maximum permissible level of metals (ppm) in poultry products set by international standards.

Elements	Types of Poultry Products				
	Eggs	Meat	Burger	Frankfurter	Liver
Essential minerals, ppm					
Iron	NA	NA	NA	NA	NA
Copper	10 [34,35]	1.0 [34]	NA	NA	1.0 [34]
Zinc	NA	20 [34,35,36]	NA	NA	20 [34,35,36]
Manganese	NA	0.5 [37]	NA	NA	0.5 [37]
Selenium	0.5 [38]	0.5 [38]	NA	NA	NA
Chromium	1.0 [34]	1.0 [34]	NA	NA	0.05 [37]
Cobalt	NA	NA	NA	NA	NA
Heavy metals, ppm					
Lead	0.50 [33]	0.10 [34]	NA	NA	0.10 [34]
Cadmium	0.05 [37]	0.05 [34]	NA	NA	0.50 [34]
Arsenic	0.1 [39]	0.1 [39]	NA	NA	NA
Nickel	NA	0.5 [39]	NA	NA	NA
Boron	10 [38]	10 [38]	NA	NA	NA
Aluminium	1 [40]	1 [40]	NA	NA	NA

NA, not available. [40], JECFA established a PTWI for Al of 1 mg/kg BW for all aluminum compounds in food.

**Table 9:** Estimated daily intake of essential elements and heavy metals per 100 g of poultry products compared to the recommended daily allowance for adults Food and Agriculture Organization/World Health Organization [FAO/WHO].

Elements	Types of Poultry Products					RDA (FAO/WHO), mg/AI	Tolerable Upper Intake Levels (U/L), mg
	Eggs	Meat	Burger	Frankfurter	Liver		
Essential elements, ppm [41]							
Iron	1.23	7.59	3.81	7.28	28.82	8	45
Copper	0.149	UDL	0.666	0.780	1.924	0.9	10
Zinc	6.29	3.85	1.49	2.64	7.98	11	40
Manganese	UDL	UDL	0.641	0.977	1.82	2.3	11
Selenium	UDL	UDL	UDL	UDL	UDL	0.55	0.4
Chromium	0.822	0.832	0.254	0.246	0.279	0.35	ND
Cobalt	UDL	UDL	0.272	0.222	0.239	ND	ND
Heavy metals, ppm [42]							
Lead	UDL	UDL	1.669	1.484	1.652	0.21	0.43 [43]
Cadmium	UDL	UDL	0.0433	0.0379	0.112	0.06	0.5-20 [44]
Arsenic	UDL	UDL	UDL	UDL	UDL	0.13	0.1 [39]
Nickel	UDL	UDL	0.710	0.728	0.668	ND [45]	0.1 [39]
Boron	UDL	UDL	UDL	UDL	UDL	NR [45]	NR
Aluminium	UDL	UDL	UDL	UDL	UDL	60 [46,47]	1 [40]

UDL = undetectable level. ND = not determined yet, RDA = recommended dietary allowances, AI = adequate intake, where no RDA has been established, but the amount is somewhat less firmly believed to be sufficient for everyone in the demographic group, UL = tolerable upper intake levels (UL). NR= no risk effect.
